# Chimpanzees (*Pan troglodytes*) strategically manipulate their environment to deny conspecifics access to food

**DOI:** 10.1038/s41598-024-68159-3

**Published:** 2024-07-30

**Authors:** Stephan P. Kaufhold, Alejandro Sánchez-Amaro, Jingzhi Tan, Sofia Fernandez-Navarro, Rebeca Atencia, Federico Rossano

**Affiliations:** 1 Department of Cognitive Science, University of California San Diego, La Jolla, CA USA; 2 Department of Comparative Cultural Psychology, Max Planck Institute for Evolutionary Anthropology, Leipzig, Germany; 3 Institute of Psychology, Leiden University, Leiden, The Netherlands; 4 Jane Goodall Institute Republic of Congo, Pointe-Noire, Congo

**Keywords:** Social evolution, Psychology

## Abstract

Humans modify their environment to grant or prevent others’ access to valuable resources, for example by using locks. We tested whether sanctuary-living chimpanzees (N = 10) would flexibly modify their environment to either allow or deny a dominant conspecific access to a shared food source by giving them the option to change a food reward’s pathway prior to releasing it. The food could end up in one of two locations: one was accessible to both the subject and a dominant conspecific, the other one was only accessible to the subject. We further manipulated the extent of inhibitory control needed for modifying the pathway by varying the subjects’ starting position. Our subjects reoriented the pathway competitively to monopolize food but changed the pathway less often in trials with high inhibitory demands. We further show how inhibitory task demands in a social context influence chimpanzees’ future planning. Our results show that chimpanzees will strategically manipulate their environment to maximize their own and deny a dominant conspecific access to food.

## Introduction

Humans intentionally adapt their environment according to their needs and goals^1^. This happens both on a collective level, for example through large-scale agriculture and architecture, but also on an individual level in everyday life, such as using a log as a bridge to cross a stream or locking ones’ door at night to prevent access by unwanted visitors. The term *affordance* describes the possible interactions an individual can have with parts of its environment^2^. Therefore, affordances are neither inherent properties of the individual, nor the environment; rather, they arise from the relationship between the two and depend on the capabilities of the individual and characteristics of the environment. Modifications of the environment can change the affordances of the modifier (log bridge example) but also affect the affordances of others (locked door example). Recognizing and manipulating the affordances of others has relevance for both cooperation and competition. Deliberate and flexible manipulations of the environment are not limited to humans and can be found in other animals, including our closest evolutionary cousins of the genus *Pan*. Chimpanzees are known to manufacture and use tools, for example to extract difficult to reach food^3–6^. However, chimpanzees’ tool use is not limited to food extraction; several studies documented the use of climbing tools in chimpanzees. For example, the classic work of Köhler^7^ demonstrated that chimpanzees can get access to out-of-reach food by using boxes and poles to climb. Similarly, Menzel^8,9^ documented the use of wooden poles as a ladder in one group of wild-born captive chimpanzees. Chimpanzees are not only capable of manipulating objects in their environment to change their own affordances, but also the affordances of others. Previous work documented that chimpanzees can also recognize and manipulate the affordances of others^10^ and can flexibly provide help according to the needs and goals of conspecifics^11^. Several studies used experimental paradigms in which chimpanzees could make environmental changes that affect conspecifics’ affordances, for example by opening doors to test their motivation for sharing food^12^, collaboration^13^, and instrumental helping^14,15^. However, it has been argued that such environmental modifications could be a by-product of the experimental design rather than revealing motivations in subjects^16^. Further, chimpanzees in an experiment by Karg and colleagues^17^ did not actively change the location of a food reward to hide it from a competitor. In the present study, we are asking whether chimpanzees will deliberately modify their environment to change the affordances of conspecifics to either gain or lose access to a mutually accessible food reward.

Members of the same group form social relationships, in which past interactions influence future ones^18^, commonly compete over resources, and adjust their behavior according to the actions of conspecifics. In naturalistic settings, sharing and competing over resources usually comes with a cost for the involved parties, for example in terms of forgoing food. When limited and valuable food is accessible to conspecifics with a social relationship, a conflict can arise with several possible outcomes. The relational model by de Waal^19^ discusses how two individuals can resolve conflicts over resources by taking the consequences for their social relationship into account. Individuals can use competitive strategies, such as aggression or theft, and cooperative strategies, such as tolerance or sharing to resolve social conflicts. Avoiding the resource or competitor has been suggested as a third alternative over competition and cooperation^19,20^. Dominant individuals have a wider range of strategies they can use to secure a resource when they find themselves in conflicts of interests with subordinates. In contrast, subordinates are more limited in their strategies. Thus, conflict avoidance is particularly relevant for subordinate individuals that are competing with dominant conspecifics. While avoiding the resource altogether prevents conflict, it also prevents the subordinate individual from benefitting from the resource. Subordinates therefore face the challenge of trying to secure resources while at the same time avoiding direct conflict with dominants^21,22^. Subordinates should assess the social context and subsequently modify their behavior or aspects of their environment according to the predicted actions, intentions, and goals of other group mates. Inhibitory control, i.e., the suppression of prepotent but ineffective responses^23^, plays a particularly important role in both securing resources and avoiding conflict in the presence of potential competitors. Beran^24^ suggested three necessary features for self-control tasks: (1) at least two different strategies must be available to the subject to solve the task, (2) different strategies need to result in different outcomes where one is clearly more valuable to the subject, and (3) obtaining the more valuable outcome must be more effortful or costly to the subject. Inhibitory control (also called self-control) is part of executive function or cognitive control, allowing individuals to avoid distractions, delay gratification and make choice aligned with their long-term interests. This cognitive ability is seen as a prerequisite for future planning, which oftentimes requires controlling immediate impulses in favor of anticipating possible outcomes^25^. For subordinates, future planning enables them to avoid impulsive actions that might lead to immediate conflict or loss of resources, make strategic decisions by anticipating the actions of dominant conspecifics, and choose strategies that minimize confrontation, such as waiting for the right moment or circumstances to act. However, the ability to plan has been highly debated in chimpanzees and other animals^25–31^. Planning for current needs can be defined as “the ability to identify an appropriate sequence of actions or consider alternative courses of action prior to execution”^32^. While great apes are documented to plan actions ahead^25^ and can modify parts of their environment step-by-step to acquire a food reward, they performed poorly in an experiment where they had to modify parts of an apparatus prior to releasing food^32^. Our study aims to address this gap by examining how chimpanzees manage inhibitory control and planning demands when they can monopolize food by strategically manipulating their environment in a social context.

Several studies have documented complex subordinate strategies to secure resources. For example, subordinates can withhold information about the location of food in the presence of dominants^33^. Many reports on tactical deception in the wild include behaviors that seem to reduce or mislead conspecifics, such as individuals concealing themselves or objects from conspecifics, or distracting conspecifics from discovering food^34,35^. Low-ranking males of different primate species have been documented to use sneak copulations, i.e., copulating with females out of sight from dominant males by seeking cover, as an alternative reproductive strategy^36–38^. Another example is the vocalizations occurring during sexual interactions between chimpanzees. One study documented that subordinate female chimpanzees will suppress copulation calls when dominant females are nearby, which might function as a strategy to avoid conflicts^39^. All these behaviors require social inhibition on the part of the subordinate.

While these observational studies do not reveal what cognitive abilities lead to the production of these behaviors, they are testimony to the ability of primates to use alternative strategies for eluding direct conflict with dominant conspecifics. A substantive range of experiments have deliberately tested cognitive proclivities and abilities that are related to flexible conflict avoidance. Many of these studies have been conducted with chimpanzees. They are of particular interest because of their phylogenetic closeness to humans and the existence of an extensive body of literature on their behavior in the wild^40^.

Chimpanzees generally perform better in competitive experimental paradigms compared to cooperative ones^41–43^. For example, one study investigated whether chimpanzees understand what conspecifics can and cannot see by placing two pieces of food in-between a dominant and a subordinate chimpanzee^44^. While the subordinate chimpanzee could see both pieces, the dominant one could only observe the placement of one piece because the other one was behind a barrier. After the food was placed, sliding doors were opened to the room with the baited food for both chimpanzees. Subordinates significantly preferred to approach the piece that only they had seen, even before the dominant started approaching the other piece. The authors described observations of spontaneous proactive strategies which were seemingly used by subordinates to avoid conflict over the food hidden from the view of the dominant. In some cases, subordinates waited to take the food until the dominant was no longer present or not facing them. In other cases, subordinates even used signals to seemingly appear to distract the dominant, such as greeting gestures and sexual offerings. Subjects in this study used the dominants’ visual attention as a cue for what they could see and used that information to predict what the dominant would do.

Many subsequent experimental designs have used similar competitive situations to examine such abilities, for example showing that chimpanzees not only understand what conspecifics can see, but also what they know^45^. This study design was similar to the previously mentioned study; however, the authors varied whether the dominant was informed, uninformed or misinformed about the location of the food item. Subjects therefore had to consider what the dominant has seen prior to the beginning of the trial. Other studies documented that chimpanzees prefer to not reveal hidden food when a competitor is present^46^ and are more likely to use a path hidden from view of a human competitor when they approach a food reward^47,48^. Results like these suggest that chimpanzees modify their behavior while obtaining food in the presence of competitors to avoid detection and thus potential conflict, which can maximize their access to resources. Predicting what others can see and know is only a useful skill in conflict avoidance as far as it helps individuals to predict what others will do. Instead of planning actions around prediction of what others will do, subordinates could also change the possible behaviors of dominants by actively modifying their environment to monopolize resources. One study tested whether chimpanzees would actively hide food to prevent a human competitor to remove their potential reward^17^. Subjects were confronted with a human experimenter that would either behave cooperatively by directly handing visible food to the subject, or competitively by taking away visible food and thus making it inaccessible for the subject. While subjects revealed hidden food to the human cooperator and kept food hidden from the human competitor, they did not modify their environment to actively hide visible food from the human competitor to maximize their reward.

**Figure 1:**
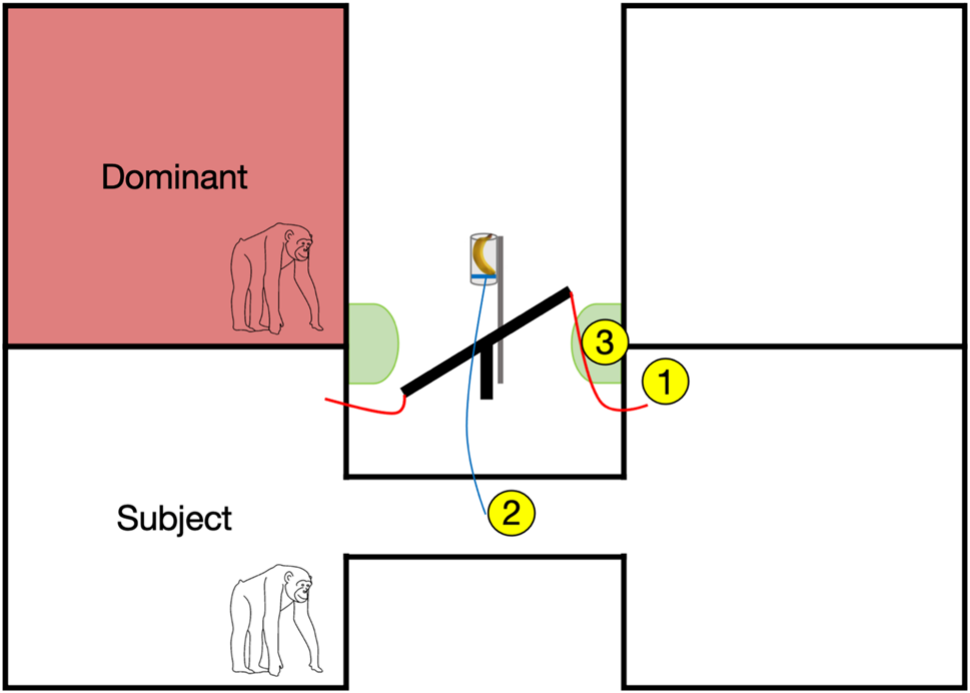
Schematic drawing of a testing session trial. This shows a mutual side trial in which the subject would have to move to the other side (high inhibitory demands) to reorient the seesaw. If the subject directly releases the food, it will end up in the location that is mutually accessible to the subject and dominant conspecific (left green area). Delivering the reward to the solo location (right green area) requires the subject to first move to the opposite room without pulling the release rope, reorient the seesaw to the right by pulling the rope attached to the right side of the seesaw (1), move back into the tunnel to now pull the food release rope (2), and subsequently collect the food from the solo food location (3).

**Figure 2:**
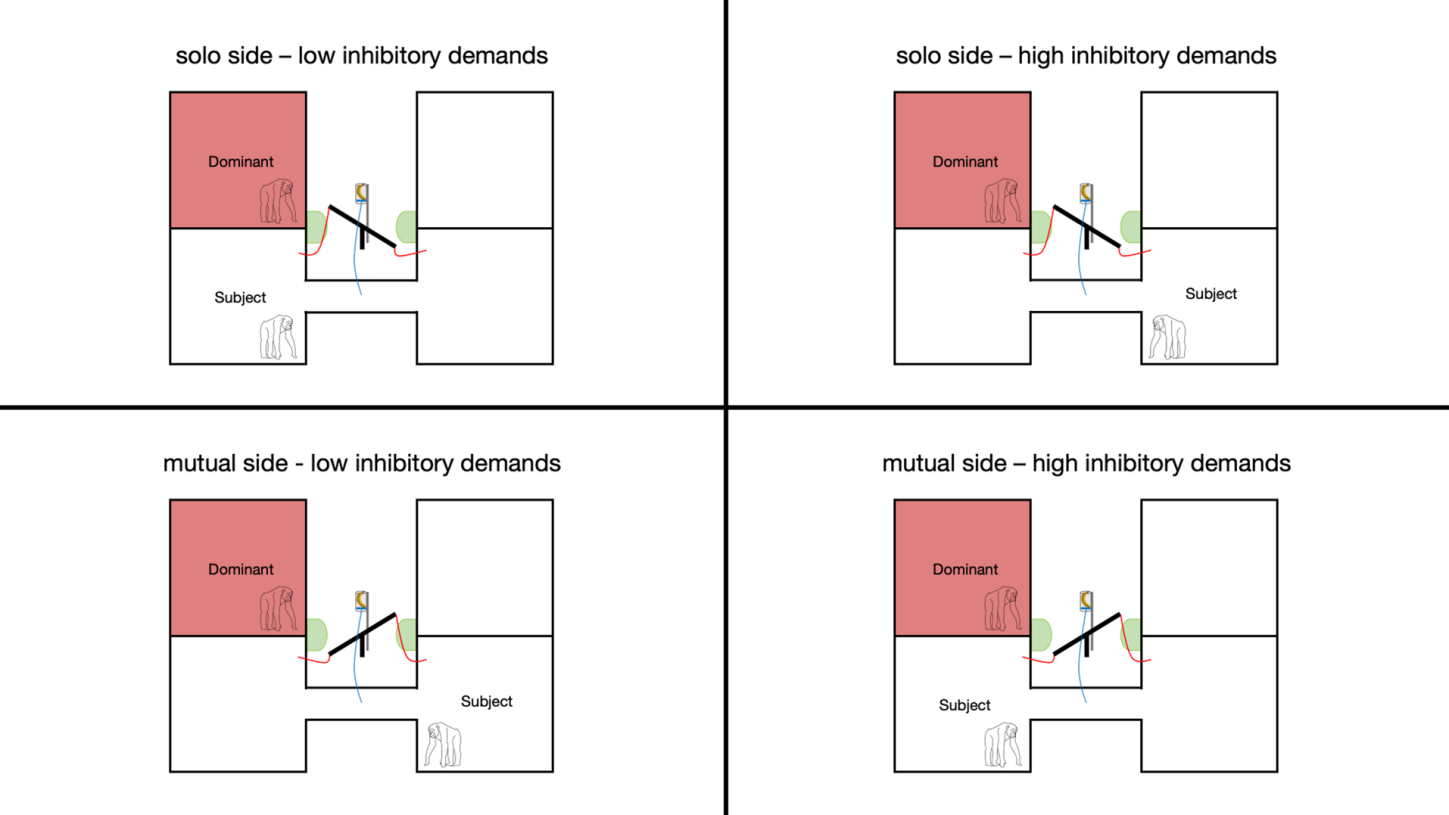
Schematic drawing of the four types of experimental trials. Example with the dominant conspecific on the left side. Each subject completed four sessions consisting of 12 trials each with 3 trials of each type. The order of trials and location of the dominant were pseudo-randomized and counterbalanced across sessions. Solo side and mutual side refer to the location the seesaw is directed to at the beginning of a trial. Low and high inhibitory demands for reorienting the seesaw are determined by the starting position of the subject, i.e., whether they must change the side and pass the food release rope to reorienting the seesaw.

Here, we presented ten dyads of chimpanzees with a novel experimental setup, consisting of a food release mechanism and a seesaw-like structure, that allowed the subordinate to flexibly manipulate the pathway of a food reward to a location that is either only accessible to them, or one that is accessible to both the subordinate subject and a dominant conspecific. Subjects could interact with the apparatus in two ways: (1) they could redirect the pathway of the food reward by pulling one of two ropes attached to the seesaw and (2) release food by pulling a rope attached to the food release (see Fig. 1). The food release mechanism by pulling a rope a. Redirecting the pathway of the food would change the affordances of their dominant conspecific: it would either lead to directing it to a mutually accessible food location or to a food location that could be monopolized by the subject. Whether redirecting the pathway would grant or deny access to the future reward depended on the initial orientation of the seesaw and the location of the dominant conspecific, which we manipulated across trials. Therefore, actively monopolizing or sharing food by reorienting the pathway required the exact same behavioral response from the subjects. Our setup thus allows to test and contrast prosocial vs. selfish motivations for changing the pathway of a food reward. We further varied the inhibitory task demands needed for reorienting the food pathway by controlling the starting position of the subject. In trials with low inhibitory demands, subjects started close to the rope allowing them to change the seesaw orientation. In trials with high inhibitory demands, subjects started on the opposite side and had pass and inhibit triggering the food release before reorienting the seesaw. The initial orientation of the seesaw, location of the dominant, and inhibitory task demands resulted in four trial types (see Fig. 2). During each trial, subject could directly release food without evaluating the pathway first. Alternatively, subjects could engage in the more effortful behavior of first evaluating the pathway and chose to either release the food or reorient the pathway first. Moreover, before releasing the reward, subjects had the opportunity to reverse their decisions of directing the food reward, providing us with potential behavioral correlates of their decision-making process.

While our task requires participants to predict what a conspecific can do, it does not require them to predict what others will be able to see such as in the study design of Karg and colleagues^17^. To monopolize or share the food reward, subjects had to first evaluate the pathway of the food and determine whether releasing the food would allow a dominant conspecific access to the food. Thus, this setup allows subjects to strategically plan for a future outcome, which could demonstrate planning abilities given that subjects could only redirect the pathway of a food reward prior to releasing it^32^. We have further varied the executive function demands required for reorienting the pathway of the food reward in our experimental design. Previous work documented that chimpanzees do not change their environment to manipulate what competitors can see, which the authors explained with inhibitory task demands^17^. To test this, we manipulated the inhibitory task demands in our study design by varying the starting position of the subject across trial. This made redirecting the food reward more effortful with regards to inhibition demands and travel distance in half of the trials. Unlike many studies on social cognition that rely on predicting what others can see, our design focuses on predicting what others can do. Additionally, subjects in our study interact with conspecifics from their group, which is more ecologically valid than interacting with a human experimenter, such as in Karg and colleagues’^17^ study.

Notably, our study design allows for a variety of different outcomes depending on the chimpanzees’ motivation and strategies. First, chimpanzees might not reorient the seesaw before releasing the food. This could indicate a lack of understanding of the apparatus or a lack of motivation in maximizing food rewards given that subjects can secure half of the rewards and can still compete over the other half directly with the conspecific. Not reorienting the seesaw could also show a willingness to donate half of the rewards to the dominant conspecific. Second, they might reorient the seesaw randomly. This might indicate that subjects did not understand the contingencies of the apparatus or do not have a consistent goal or preference. Third, subjects might significantly more often reorient the seesaw towards the mutual side. An explanation for this behavior could be a prosocial motivation to either donate the food to the dominant conspecific in case the subject will not obtain rewards themselves, or a preference for sharing and co-feeding with the dominant conspecific, similar to the findings of an experimental study in which bonobos shared food rewards with an unfamiliar conspecific as long as they had the ability to socially interact in the same room^49^. Thus, directing the food rewards towards the location that both subject and conspecific could access would suggest a cooperative or prosocial motivation. Fourth, subjects could show a preference for reorienting the seesaw towards the location that only they can access. This modification of the food pathway would prevent the dominant conspecific from being able to access the food reward and thus would indicate a preference of the subject for maximizing their own payoff by avoiding direct conflict over food. We hypothesize that chimpanzees will manipulate the food pathway to render it inaccessible to the dominant conspecific and thus monopolize the reward. We further predict that subjects are more likely to redirect the pathway when inhibitory task demands are lower. Lastly, we predict that subjects are more likely to redirect the food pathway when the reward is indivisible compared to a divisible reward.

## Results

**Figure 3:**
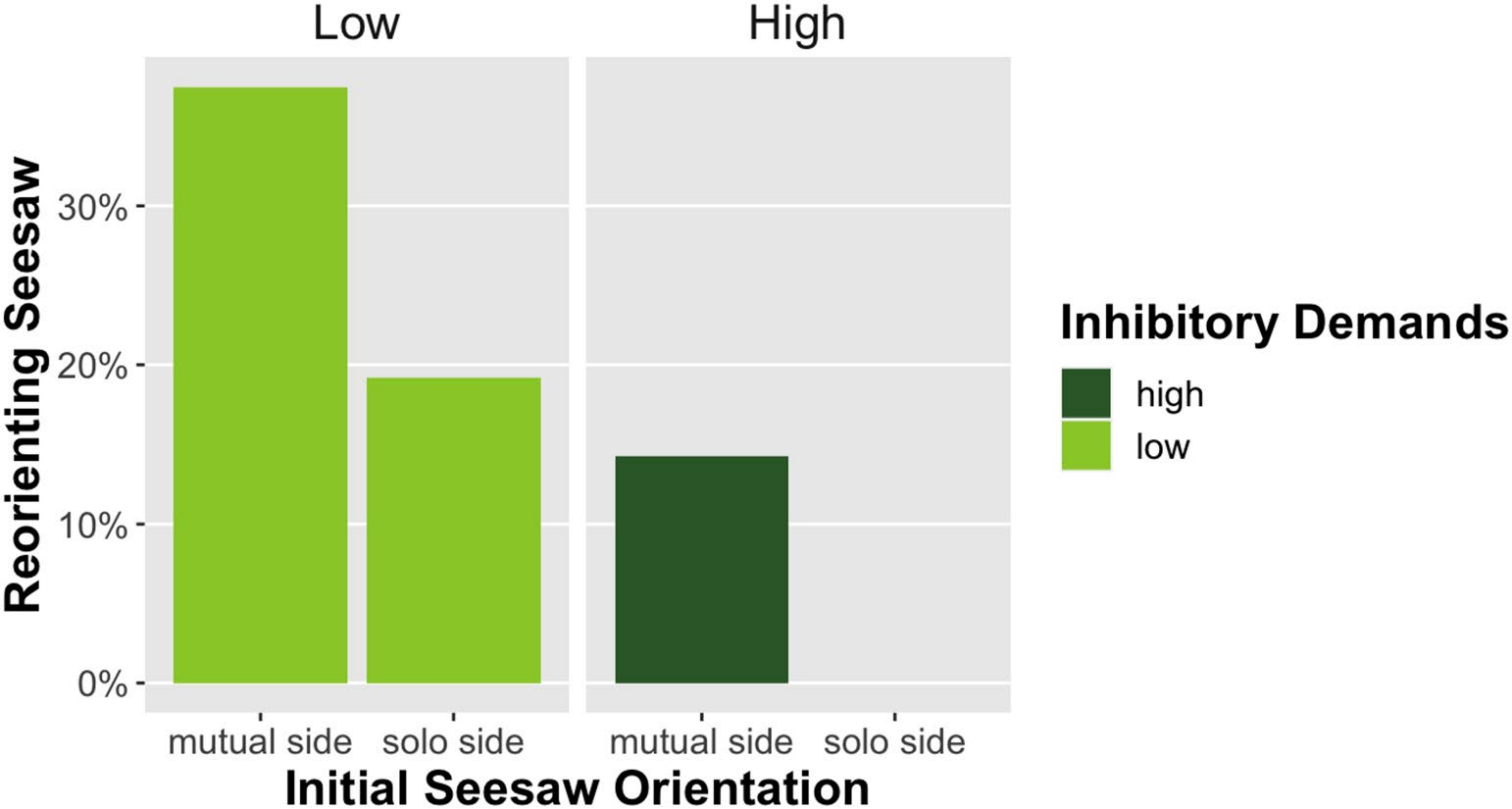
Percentage of reorienting the seesaw for different initial seesaw orientations at the beginning of a trial (mutual side vs. solo side) and inhibitory demands (high vs. low) operationalized by the subjects’ starting position.

Chimpanzees in our study flexibly changed the pathway of a food reward to deny their conspecific access. Subjects reoriented the seesaw in 25.9% of mutual side trials compared to 9.6% in solo side trials. Thus, they redirected the food reward significantly more often when it would change the food pathway from a location that was mutually accessible to them and a dominant conspecific, i.e., the mutual side, to a location that was only accessible to themselves, i.e., the solo side (Model 1: OR: 5.56; df = 7, p < 0.01*, N = 479). Further, subjects were significantly more likely to reorient the seesaw when they started close to the side that allowed reorienting (28.4%), requiring lower inhibitory control, compared to when they started on the opposite side (7.1%), where they had to pass the food release mechanism before reorienting the seesaw, thus demanding higher inhibitory control (Model 1: OR: 9.00; df = 7, p < 0.01*, N = 479). During trials with a far starting position (high inhibitory demands), subjects only reoriented the seesaw when it was directed towards the mutual side at the beginning of the trial (see Fig. 3). We found the same patterns when only the trials of the first session of each subject were considered (Model 2). Two subjects (Ngoro, Isabelle) never reoriented the seesaw (see Table S1). Our manipulation of inhibitory control therefore shows how inhibitory task demands affect chimpanzees’ behavior in a social context. While the majority (N=8) of subjects would at least sometimes reorient the seesaw in low inhibitory demand trials, only a minority (N=4) reoriented the seesaw in high inhibitory demand trials (see Table S1). We further observed release inhibition behavior, i.e., instances in which subjects would first touch but then inhibit pulling the *food release rope* in 5% of trials (22/479). While these instances were not many in number, they were in line with the expected motivation and inhibitory challenges observed of reorienting the seesaw across the four different trial conditions (Table S2). Release inhibition occurred most frequently in trials where the initial pathway of the food was towards the mutual side. Subjects also engaged in redundant or ineffective seesaw rope pulling, which we defined as touching or pulling the rope attached to the lowered end of the seesaw prior to releasing the food. Chimpanzees touched or pulled on the ineffective rope in 14% (68/479) of trials. This redundant action occurred predominantly in trials with high inhibitory demands (N=67) and only once in a low inhibitory demands trial. The initial orientation of the seesaw did not seem to play a role for redundant rope pulling, with 36 (53%) instances occurring in solo side and 32 (47%) instances in mutual side trials. Additionally, we observed three trials in which subjects first reoriented the seesaw, then inhibited releasing the food and reoriented the seesaw again to the initial position at the beginning of the trial. All these trials (Mambou: 2, Zola: 1) were low inhibitory demands trials with the initial pathway pointing to the solo side. In terms of actual rewards obtained by the subjects and competitors, reorienting the seesaw away from the mutual side did on average increase the rewards for the subjects and decrease the rewards for the conspecific for both divisible and non-divisible rewards (see Table 1). The inverse was true for reorienting the seesaw away from the solo side. When subjects directly competed over the food reward with the conspecific in trials where the seesaw was not reoriented and directed towards the mutual side, the dominant conspecific did on average obtain more food than the subject. This confirmed that the conspecific was indeed dominant over the subjects and denying the dominant access was an effective strategy to obtain more food. However, subjects still on average obtained some rewards in these trials, showing that they were willing to physically compete over food rewards with their conspecifics.

**Table 1:** Mean amount and standard deviation (SD) of food pieces obtained by subject and conspecific in mutual and solo orientation trials and when reorienting vs. not reorienting the seesaw. The apparatus was baited with one piece of banana in the non-divisible reward condition and five peanuts in the divisible reward condition.

Reward	Initial orientation	Reorienting	Subject	Conspecific
Divisible	Mutual	No	0.25 (SD = 0.44)	0.75 (SD = 0.44)
*1 Banana*	Solo	No	0.93 (SD = 0.26)	0.01 (SD = 0.10)
	Mutual	Yes	0.97 (SD = 0.18)	0 (SD = 0)
	Solo	Yes	0.55 (SD = 0.52)	0.45 (SD = 0.52)
Non-divisible	Mutual	No	1.83 (SD = 1.28)	2.38 (SD = 1.4)
*5 Peanuts*	Solo	No	3.54 (SD = 1.19)	0 (SD = 0)
	Mutual	Yes	3.73 (SD = 1.05)	0.1 (SD = 0.54)
	Solo	Yes	2.17 (SD = 1.34)	1.75 (SD = 1.36)

### Model 1: Performance across all sessions

To test predictors for reorienting the seesaw, we ran a generalized mixed model), which included seesaw orientation (solo/mutual), inhibitory demands (low/high), reward type (peanuts/banana), conspecific side (left/right), session number, subject age and sex as fixed effects and subject identity as a random effect. The full model (Table S3) was significantly better in predicting reorienting of the seesaw compared to the null model which only included the random effect (χ^2^ =85.55, df = 7, p < 0.01*). Both the initial seesaw orientation (OR: 5.56; df = 7, p < 0.01*, N = 479) and starting position of the subject (OR: 9.00; df = 7, p < 0.01*, N = 479) significantly predicted reorienting. Given the absence of reorienting in this condition, our models could not include the interaction between the initial orientation of the seesaw and inhibitory demands. There was a trend towards significance for sex of the subjects, with males reorienting the seesaw more often than females (OR: 7.36; df = 7, p = 0.07, N = 479). Contrary to our predictions, reward type did not significantly predict the likelihood of reorienting the seesaw, i.e., subjects did not change their behavior in trials with highly monopolizable food compared to divisible food. Conspecific side, session number, and age of the subject did not increase the likelihood of reorienting the seesaw.

### Model 2: First session performance

To control for learning effects and analyze subjects’ spontaneous behavior, we assessed performance during their first session only in a second generalized linear mixed model. We included seesaw orientation (solo/mutual), inhibitory demands (low/high), and reward type (peanuts/banana) as fixed effects and subject identity as a random effect. Again, the full model (Table S4) was significantly better in predicting seesaw reorientations compared to the null model only including the random effect (χ^2^ = 22.93, df = 3, p < 0.01*). Subjects were significantly more likely to reorient the seesaw during trials with a mutual side initial seesaw orientation compared to a solo side initial orientation (OR: 7.48, df = 3, p < 0.01*, N = 120). Further, they were significantly more likely to reorient the seesaw during trials with a close starting position (OR: 7.48, df = 3, p < 0.01*, N = 120). Therefore, we found the same significant factors predicting reorienting behavior as in the first model, i.e. subjects’ behavior did not change across sessions. This indicates that our subjects’ performance is not explained through a learning effect across sessions. There was no significant effect for food reward.

## Discussion

Chimpanzees in our study strategically monopolized food rewards by modifying their environment to deny a conspecific access to a future reward. Rather than actively modifying the food pathway cooperatively to actively co-feed or donate rewards to a dominant conspecific, our results suggest that on a group level subjects’ reorienting was motivated by monopolizing food rewards. They thus reoriented the pathway to maximize their reward by denying the dominant access. By keeping the actions required for achieving competitive and cooperative outcomes the same across trials, these findings cannot be explained as a simple by-product of task design. Chimpanzees did not indiscriminately pull the first rope that could reorient the seesaw. Rather, their behavior was indicative of their competitive goals and motivation to monopolize resources by preventing food access to the conspecific. They have learned two possible ways to interact with the apparatus during the training (reorienting the seesaw and releasing food) and applied these skills flexibly depending on the initial pathway of the food reward and location of the dominant conspecific. They overall inhibited reorienting the seesaw to the mutual side. Further, our results are unlikely to be explained by conditional discrimination learning^50^ because this pattern was already observable during first session trials. Rather, this finding suggests that our subjects were able to engineer their environment in a way that let them monopolize the future reward while avoiding direct resource conflict with the conspecific, suggesting a higher-level cognitive process at play. To monopolize rewards, our subjects did not have to manipulate what a conspecific could *see,* such as in the study by Karg and colleagues^17^, instead they had to anticipate and manipulate what others could *do*. This could suggest prospective abilities or future planning in our subjects because they had to identify an appropriate sequence of actions based on the state of the apparatus prior to releasing the food^32^. First, they had to predict the pathway of the food reward and anticipate possible behavioral responses of their conspecific, and subsequently either reorient the seesaw or inhibit doing so to manipulate the possible actions of the dominant and thus monopolize the reward. While subjects in Karg and colleagues’^17^ study did not actively hide food from a competitor to monopolize it, we found that our subjects would monopolize food by actively preventing their conspecific from gaining access to it. Similar to the experimental design of Tennie and colleagues^16^, performing the same action sequence, i.e., reorienting the seesaw, could result in either access to or denial of food for the conspecific depending on their location. However, our design differed regarding the outcome to the subject because their actions could affect their own access to food. Therefore, our design allowed to directly test cooperative against competitive motivations with food at stake for both the subject and conspecific, resulting in a zero-sum game in terms of food rewards. Subjects were less likely to reorient the seesaw during trials where it was initially oriented towards the mutual side and they started the trial opposite of the side that allowed reorienting it, i.e., when they had to pass the food releaser and had to inhibit pulling it before reorienting the seesaw. This shows how interindividual differences in inhibitory control can affect the ability to employ competitive strategies. Notably, we only observed reorientations of the seesaw during high inhibitory control trials that would result in redirecting the reward to the solo location and never to the mutual location.

Our results further show how inhibitory control affects subordinate chimpanzees’ ability to strategically compete with a dominant conspecific. Higher inhibitory control allowed subordinate subjects to maximize their food rewards by reorienting the seesaw when it was directed towards the mutual side. While most subjects (8 /10) used reorienting as a strategy to maximize their payoff in the condition with lower inhibitory demands, only four did so in the condition that required higher inhibitory demands. Chimpanzees in our study sometimes touched the food release rope, inhibited pulling it and instead reoriented the seesaw first before releasing the food. This occurred most often in trials where the initial seesaw orientation was towards the mutual side and where subjects needed high inhibitory control to reorient the seesaw because they had to pass the food release to do so. One explanation for this behavior is that they inhibited their initial impulse to immediately release the food when they evaluated the state of the seesaw and position of their conspecific while they were in the overhead tunnel with the release. This is reminiscent of the hand wavering behavior of chimpanzees in a touch screen task recently reported^51^. In this study, chimpanzees were more likely to hand waver across different touch screen choices in a transitive inference task when the choices were more difficult. Subjects also touched or pulled the ineffective seesaw rope that would not change the orientation during some trials. This predominantly occurred in trials high with inhibitory demands, suggesting that this condition imposed increased cognitive challenges such as inhibiting prepotent behaviors or task loading. Unlike most experimental designs on cooperation and competition in primates, our seesaw setup allowed subjects to reverse their decision of orienting the seesaw prior to releasing the food. While this happened only during three trials, all of those occurred in the same type of trial, i.e., a trial with low inhibitory demands with the initial orientation of the seesaw towards the solo side. This reversal of the modification of the pathway is aligned with the overall pattern that we saw, i.e., subjects’ tendency to release food towards the location only they could access.

Contrary to our prediction, chimpanzees were not more likely to reorient the seesaw towards the solo side in trials with non-divisible food rewards (one piece of banana) versus divisible food rewards (five peanuts). One possible explanation for this is that the perceived value of both types of rewards could have been too close to elicit different behavioral responses. Drastically increasing the number of peanuts in the divisible reward condition could have led to a higher willingness of the subjects to co-feed and not reorient the food to the solo side.

In addition to maximizing rewards, subjects also could have redirected the reward to the location only accessible to them to avoid direct conflict and physical altercations with a dominant conspecific. Our experimental design did not allow us to tease apart whether subjects reoriented to maximize their food reward or whether they avoided competition over the reward with the dominant conspecific. Future studies could investigate this by creating a condition under which having direct competition over a food resource with a conspecific would lead to a higher reward to the subject than avoiding food competition by feeding from another location. Alternatively, subjects could be partnered with a subordinate conspecific or friend.

One alternative explanation is that subjects reoriented the seesaw towards the solo side to maximize the distance to the dominant conspecific. Preferring to eat far away from dominant conspecifics might be a common strategy to avoid direct conflict. However, we do not think an aversion to the mere presence of the dominant conspecific can explain our observed results given that subjects would still directly compete dominants when it was delivered to the mutual side and indeed obtain some fraction of the rewards.

Our findings highlight the ability of subordinate individuals to acquire resources through strategically making use of their environment to monopolize food and avoid direct conflicts with dominants by actively denying access to food. The ability to predict what resources could be accessed by others and then strategically rendering them inaccessible to others might be seen as a precondition for establishing ownership without the use of force, dominance, or aggression. This is a common way of protecting resources in humans, for example by building fences or using locks. While great apes respect possessions of conspecifics, there is currently no evidence for respecting ownership^52^. Chimpanzees do not appear to build such structures in the wild and might have few opportunities to prevent conspecific’s physical access to locations. Nonetheless, our study suggests that they have the cognitive ability to use preexisting structures to this end.

## Materials and methods

### Subjects

Ten chimpanzees (*Pan troglodytes*, 4 females; mean age 10.3 years) located at the Tchimpounga Chimpanzee Sanctuary (Republic of Congo) completed the current study (see Table 2). One additional male chimpanzee (Motambo, 12 y/o) participated in the study as a dominant conspecific for the most dominant subject (Mambou). All chimpanzees arrived at the sanctuary as orphans and were housed in the same group (Tchindzoulou) on an island on the Kouilou river with a dense tropical forest of about one square kilometer in size. All our tests took place within their dormitory structure where they had *ad libitum* access to water. Our studies did not interfere with their regular feeding schedule. This study adhered to the ethics protocols of the University of California, San Diego and all data collection in the Tchimpounga Natural Reserve was approved by the Jane Goodall Institute and local authorities (permit no. 005/MRSIT/DGRST/DMAST). All methods are reported in accordance with ARRIVE guidelines (https://arriveguidelines.org). This includes detailed descriptions of study design, sample sizes, randomization, blinding, and statistical methods to ensure transparency and reproducibility.

**Table 2:** Subject details. Sex, age, and dominant conspecific during testing of all participating subjects.

Subject	Age	Sex	Dominant Conspecific
Alex	9	Male	Mambou
Dunez	9	Female	Mambou
Isabelle	14	Female	Makazi
Leki	10	Male	Makazi
Makazi	11	Male	Mambou
Mambou	10	Male	Motambo
Moukolo	11	Female	Makazi
Ngoro	15	Female	Mambou
Willy	6	Male	Mambou
Zola	8	Male	Mambou

### Apparatus

**Figure 4:**
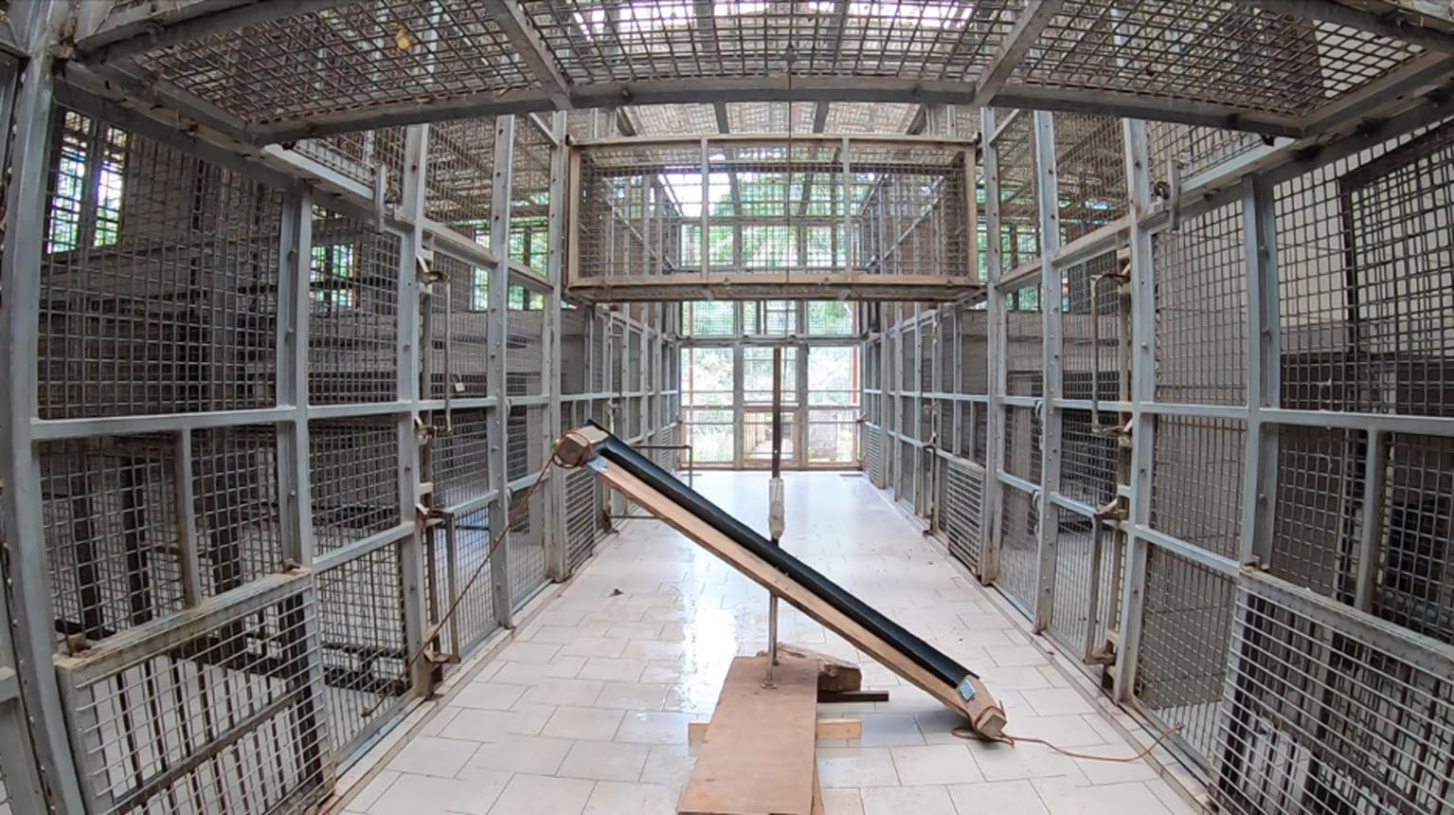
Picture of apparatus setup. Taken from the video camera’s point of view in the hallway. Subjects could move between the left room in the front and right room in the front using the overhead tunnel. Pulling the rope attached in the middle of the subject’s overhead tunnel would release food that would then fall on the seesaw and slide down. The picture shows the seesaw oriented to the right side. In this constellation, released food would fall to the right side by default; however, the orientation of the seesaw could be manipulated by pulling the rope on the left side, which would change the reward pathway to the left side. The apparatus was placed in-between the rooms of the subject and the room in which a dominant conspecific was placed during test sessions. Food that would slide down the seesaw to the right side would be accessible from the close room on the right side and the adjacent room on the right side and vice versa on the left side.

Our apparatus consisted of two functional parts: a food release mechanism and a seesaw-like structure (hereafter seesaw) that formed the pathway the food would take after being released (see Fig. 4). The apparatus was placed in the middle hallway that separated the sleeping rooms on the left side from the sleeping rooms on the right side. The hallway was inaccessible to chimpanzees. However, overhead fenced runways connected the right and left side of the sleeping rooms and could be opened or locked by the animal caretakers. The seesaw directed the released food either to the left side or right side, depending on its orientation. Holes in the fence (10 × 10 cm) allowed chimpanzees to access food rewards from both rooms on each side.

### Food release mechanism

The food release mechanism consisted of a transparent plexiglass tube (diameter: 6 cm, height: 21 cm) that was fixed on a metal rod with two legs. A piece of cardboard could be inserted into a slit in the tube, resulting in a closure of the tube’s bottom opening. Food rewards could be placed inside the tube on top of the cardboard, allowing the subject (and conspecific in test sessions) to see the potential food rewards. The tube was positioned above the center of the seesaw. The cardboard was tied to a rope that was connected to a fenced overhead tunnel on the other end via a U-bolt. Pulling the rope removed the cardboard from the tube, leading to the baited food dropping down onto the seesaw.

### Seesaw

A structure reminiscent of a seesaw was placed in-between the hallway separating the left rooms from the right rooms in the dormitory (see Fig. 4). The seesaw was built using a wooden plank (length: 2 m, width: 31 cm) which was connected to a U-shaped metal arch on top of a wooden base (height: 63 cm) using U-bolts. A halved PVC tube was placed on top of the wooden plank. A rope was attached on each end of the seesaw on a wooden block and was connected via a U-bolt to the nearby room of the subject. Both ropes were attached to the board by pulling them through and wrapping them around a hole in the wooden block at the end of the plank on each side. Pulling the rope would change the orientation of the seesaw depending on its position. Additional weights were placed on the base of the seesaw to reduce movement caused by subjects forcefully pulling on the attached ropes.

### Procedure

Subjects completed all training sessions and experienced feeding competition with their conspecific before they began with the four testing sessions.

### Apparatus training

All subjects completed four different training conditions in the same order before they participated in test sessions. Each training condition was completed as soon as the subject succeeded in either six consecutive trials, or seven out of eight trials. We used five peanuts as a reward in all training trials.

### Release training

The goal of the first training was to familiarize subjects with the food release mechanism. The orientation of the seesaw (left/right) was pseudorandomized across trials and subjects could not change the orientation. Subjects could only release the food reward by pulling the rope attached in the middle of the overhead tunnel. A trial was considered successful if the subject released food by pulling the rope and subsequently entered the room with access to the reward first within three minutes.

### Balanced seesaw

The goal of this training was to familiarize the subjects with the possibility to orient the seesaw to either the right or left side. The setup was the same as in the release training, with the exception that the seesaw was balanced, i.e., parallel to the floor, at the beginning of each trial and had one rope attached to each end, enabling the subject to change the orientation from either the left or the right room. If the subject would release the food first, it would drop onto the seesaw and remain in the middle, which helped to draw the subject’s attention to the seesaw and the ropes attached to it. To retrieve the reward, the subject had to release the food and pull on one of the ropes attached to the seesaw in any order. Subjects could also change the orientation of the balanced seesaw to either side before releasing the food. A trial was considered successful if the subject delivered and subsequently accessed the reward to one of the two sides within three minutes.

### Turned-away seesaw

Each subject completed two sessions of this training in which they had access to the tunnel and either the left or the right room (counterbalanced). Access to the alternative room was blocked by locking one door at the end of the runway that would connect to the other room. The goal of this training was to make subjects sensitive to the order in which they reoriented the seesaw and released the food. The seesaw was always directed away from the room the subject had access to. Therefore, releasing food before reorienting the seesaw delivered the food to the inaccessible location. The experimenter did not remove food that ended up in the inaccessible location across trials of the same testing day to make the lost reward more salient to the subjects. Thus, subjects had to learn to first change the orientation of the seesaw, and only subsequently release the food reward. A trial was considered successful if the subject first reoriented the seesaw and subsequently released the food within three minutes.

### Reorienting inhibition training

The setup is the same as in the release training with the exception that ropes are attached at both ends of the seesaw to allow for reorienting. Again, the initial orientation of the seesaw was determined by pseudo-randomization. Subjects would always start a trial on the opposite site of where the seesaw was oriented to. The goal of this training is to ensure that subjects do not default to always reorienting the seesaw towards the room they started the trial in and to introduce them to the apparatus setup of the test sessions. Not reorienting the seesaw prior to releasing the food is the more efficient strategy in this scenario given that the subject is of equal distance from both potential food locations when triggering the release mechanism. Thus, reorienting the seesaw is an unnecessary step in this training. A trial was considered successful if the subject releases the reward without reorienting the seesaw within three minutes.

### Introduction of dominant conspecific

These trials had two goals: first, subjects were introduced to the presence of a dominant conspecific that had equal access to food on one side in a room adjacent to the subject; second, the conspecific was introduced to the ability to obtain food from the apparatus. Every subject was paired with a dominant conspecific. The dominant conspecific remained constant for each subject across sessions. We chose each dominant conspecific with the help of six animal caretakers, who were independently questioned about the dominance relationships of all pairs used in this study and showed perfect agreement. Two dominant conspecifics (Makazi, Mambou) did also participate as subjects in this study. The third (Motambo) did not participate as a subject and was only paired with Mambou, the chimpanzee that was rated most dominant out of the individuals participating in our experiment. Dominant conspecifics were put in either the left or right room adjacent to the subject’s room. The location of the dominant conspecific during the introduction was counterbalanced across subjects. Both rooms which could contain the dominant conspecifics had the same openings in the fence as in the subject rooms, allowing the conspecific to reach for one of the two possible reward locations. Subjects were placed in the room adjacent to the dominant conspecific and access to the tunnel and other room were blocked. The seesaw was directed towards the side of both the dominant and the subject (equal to *mutual* side in test condition described below) and the ropes on the seesaw were detached. The experimenter released food (5 peanuts) from the feeder by hand while both subject and dominant conspecific were within reach of the mutual reward location. Every subject experienced three consecutive trials right before the beginning with the first test session. A trial was considered successful if both chimpanzees attempted to collect peanuts. Attempts to collect the food reward were operationalized by chimpanzees putting their hands through the cut-out opening in the fence that allowed them to access the food location. On average, subjects obtained 2.1 peanuts (SD=1.05) and conspecifics 2.4 peanuts (SD=1.13) per trial. Some peanuts fell out of reach of both the subject and conspecific. The experimenter removed those peanuts after the end of a trial.

### Test sessions

The general setup was the same as in the reorienting inhibition training with the exception that a dominant conspecific was placed in either the left or right room adjacent to the subject (see Fig. 1). During each session, the conspecific only had access to one room while the subject could access both sides. The side of the conspecific was counterbalanced across all four sessions. We refer to the side with the dominant conspecific as the *mutual* side, because both the subject and the conspecific could access the food reward on that side. The opposing side is referred to as the *solo* side because the conspecific was not able to access the food reward from his room. Thus, food on the solo side was only accessible to the subject. Counterbalancing the location of the dominant conspecific therefore served as a control condition to ensure that reorienting the seesaw was not a simple by-product of the task design. We also manipulated the reward across sessions: one half of subjects started with bananas for the first two sessions and ended with peanuts and the other half of subjects had the reverse order. In the banana reward condition, the apparatus was baited with one single piece of banana. The reward was of high value to the subjects and was highly monopolizable, i.e., it could not be shared between the two chimpanzees. In the peanut reward condition, the apparatus was baited with 5 peanuts. Peanuts were a desirable food reward for chimpanzees and unlike bananas could lead to a split distribution between subject and dominant if they dropped to the mutual side. We therefore predict that subjects should be more likely to reorient the seesaw during mutual trials that use bananas as a reward. Each session consisted of twelve trials. Therefore, each subject completed 48 trials in total. The seesaw orientation at the beginning of each trial was pseudorandomized within each session. Half of the trials within a session started with the seesaw oriented towards the *mutual* side, the other half started with the seesaw oriented towards the *solo* side. Hence, if subjects want to monopolize the food reward, they should only reorient the seesaw during trials with the seesaw oriented towards the mutual side. Delivering food to the mutual side was not synonymous with losing it; the subject still had a chance to acquire the food but had to share or directly compete over it with the dominant conspecific. Therefore, if subjects do not reorient the seesaw at all, they still can monopolize half of the rewards and compete directly over the other half. We manipulated the starting position of the subject at the beginning of each trial by donating food (1 peanut) to them either on the left side of their left room or right side on their right room. This was an operationalization for inhibitory task demands. The starting position was *close* if the subject began the trial in the room from which the seesaw could be redirected directly. Thus, reorienting the seesaw in the close position was equivalent to *low* inhibitory task demands. In contrast, the *far* starting position meant that the subject could only reorient the seesaw by passing through the tunnel and moving to the other room. Note that subjects had to pass the release mechanism in the tunnel and inhibit releasing food to reorient the seesaw in *far* starting position trials. Therefore, reorienting the seesaw demanded *high* inhibitory control from the subjects in far trials. The combination between the initial seesaw orientation (*mutual/solo*) and starting position of the subject (*low inhibitory demands/high inhibitory demands*) resulted in four different trial types: solo-low inhibitory demands, solo-high inhibitory demands, mutual-low inhibitory demands, and mutual-high inhibitory demands (see Fig. 2). A test trial began as soon as the seesaw was oriented by E1 (measured by the point in time at which the lowered side of the seesaw was touching the floor) and ended as soon as the subject released all food or after two minutes had passed. A trial was repeated up to five times if the subject did not trigger the release mechanism within two minutes. Each subject participated in no more than one testing session per day. We used four different pseudo-randomizations to determine the seesaw orientation and starting position of the subject for each session. Each randomization included three trials of every of the four trial types described above.

### Scoring and statistical analysis

All trials have been video recorded using one wide-angle camera (GoPro Hero7) capturing the experimental setup. S.K. coded all instances of reorienting the seesaw, releasing, accessing, and collecting food across all trials. Notably, subjects could change the orientation of the seesaw multiple times within a trial prior to releasing the food as long as they did so within the trial duration limit of two minutes. Reorienting the seesaw twice would result in the same orientation as at the beginning of the trial. For our models, this was counted as if the subjects had not reoriented the seesaw. However, we provide the numbers for these reorienting reversals in the descriptive results section. Besides reorienting the seesaw and releasing food, we also coded instances in which subjects would touch food release rope without pulling it strong enough to trigger the release (release inhibition). We also coded instances of ineffective and redundant rope pulling on the seesaw, i.e., instances of subjects touching or pulling the rope of the seesaw attached to the lowered side before releasing the food, which did not reorient the pathway. We further coded the amount of food that both the subject and conspecific collected during each trial. The delay between releasing food and trying to access the reward by extending the arm through the hole in the fence was further coded in seconds. A second coder, blinded to the hypotheses, scored the same variables for 20% of the trials (N=96) to assess inter-rater reliability. We used Cohen’s kappa for assessing the agreement between raters for categorical variables and intraclass correlation (ICC) for the amount of food pieces that the subject and conspecific obtained. The agreement was almost perfect^53^ for categorical variables (reorienting seesaw: k=0.97; inhibition pulling: k=0.92; and excellent^54^ for the quantity of food obtained by the subject (ICC: 0.98) and the conspecific (ICC: 0.98). We further coded the time that passed between a subject releasing the food by pulling the rope attached to the tube containing the reward and the subject putting their hand through the opening of the fence that allowed them to access the reward location. One trial (Makazi: mutual-high inhibitory demands) was lost due to an experimenter error, resulting in 479 trials across all subjects in total. All statistical analyses have been conducted using R 3.4.2^55^. Using the ‘glmer’ function of the ‘lme4’ package^56^, we ran two generalized linear mixed models (GLMMs) with binomial distributions to assess which factors would influence reorientation of the seesaw in our experiment. Our first model (Model 1) included all sessions for each subject (479 data points in total). We included seesaw orientation (solo/mutual), inhibitory demands(low/high), reward type (peanuts/banana), conspecific side (right/left), session number (1/2/3/4), age of the subject, and sex of the subject (female/male) as fixed effects. Subject identity was included as a random intercept. The goal of the second model was to assess subjects’ performance during their first session to control for learning effects and analyze their spontaneous behavior in a novel situation. For Model 2, we only considered the performance during the first session across all subjects, resulting in 120 data points. We included the seesaw orientation (solo/mutual), inhibitory demands(low/high), and reward type (peanuts/banana) as fixed effects. Again, subject identity was included as random intercepts in the model. We were not able to include the interaction between the initial seesaw orientation and inhibitory demands in our models given that there were zero instances of reorienting the seesaw in high inhibitory demand trials with the initial orientation of the seesaw towards the solo side. We compared both full models to their corresponding null models using ‘Chisq’ from the ‘anova’ function in R to determine whether they were significantly better at predicting the reorientation of the seesaw within a trial.

### Ethical approval

This study adhered to the ethics protocols of the University of California, San Diego and all data collection in the Tchimpounga Natural Reserve was approved by the Jane Goodall Institute and local authorities (permit no. 005/MRSIT/DGRST/DMAST). All methods are reported in accordance with ARRIVE guidelines (https://arriveguidelines.org). This includes detailed descriptions of study design, sample sizes, randomization, blinding, and statistical methods to ensure transparency and reproducibility.

### Supplementary Information

Supplementary Tables.

## Data Availability

All data and code used for analyses of this study are available on Dryad: 10.5061/dryad.5tb2rbp9w.
