# Supplementary material for: Chimpanzees (Pan troglodytes) strategically manipulate their environment to deny conspecifics access to food
Source: Sci Rep. 2024 Jul 30;14:17579. doi: 10.1038/s41598-024-68159-3 (PMC11289288; doi:10.1038/s41598-024-68159-3)
Supplement: Supplementary file 1 — Supplementary Tables. [file 41598_2024_68159_MOESM1_ESM.docx]

Supplementary Materials for

**Chimpanzees (*Pan troglodytes*) strategically manipulate their environment to deny conspecifics access to food**

Kaufhold *et al.*

**This file includes:**

Tables S1 to S4

| **Table S1.** | | | | | | |
| --- | --- | --- | --- | --- | --- | --- |
| *Total number and percentage of seesaw reorientations for different initial seesaw orientations and starting positions for each subject.* | | | | | | |
|  | **Mutual Side** | | | **Solo Side** | | |
| **Inhibitory Demands** |  | *Low* | *High* |  | *Low* | *High* |
| **Subject** |  |  |  |  |  |  |
| Alex |  | 11 (91.7%) | 5 (41.7%) |  | 4 (33.3%) | – |
| Dunez |  | 3 (25%) | 1 (8.3%) |  | 2 (16.7%) | – |
| Isabelle |  | – | – |  | – | – |
| Leki |  | 1 (0.8%) | – |  | 4 (33.3%) | – |
| Makazi |  | 10 (83.3%) | 5 (45.4%) |  | 8 (66.7%) | – |
| Mambou |  | 8 (66.7%) | – |  | 1 (8.3%) | – |
| Moukolo |  | 2 (16.7%) | – |  | 3 (25%) | – |
| Ngoro |  | – | – |  | – | – |
| Willy |  | 4 (33.3%) | – |  | – | – |
| Zola |  | 6 (50%) | 5 (41.7%) |  | 1 (8.3%) | – |
| TOTAL |  | 45 (37.5%) | 16 (13.4%) |  | 23 (19.2%) | – |

| **Table S2.** | |
| --- | --- |
| *Percentage and absolute numbers of subjects redirecting the seesaw to its initial position after inhibiting to pull the food release rope across different trial types.* | |
| **Condition** | **Redirecting Seesaw after Release Inhibition** |
| Mutual-high | 83% (10/12) |
| Mutual-low | 74% (3/4) |
| Solo-high |  |
| Solo-low | 17% (1/6) |
| Total | 64% (14/22) |

| **Table S3.** | | | | |
| --- | --- | --- | --- | --- |
| *Model 1: Seesaw reorientation predictors across all sessions.* | | | | |
| **Factor** | **Odds Ratio** | **Lower CI** | **Upper CI** | **P-value** |
| Intercept | 0.02 | 0 | 6.79 | 0.2 |
| Seesaw Orientation | 5.56 | 2.91 | 10.59 | < 0.01 *** |
| Starting Position | 9.00 | 4.52 | 17.89 | < 0.01 *** |
| Reward | 0.94 | 0.52 | 1.72 | 0.85 |
| Conspecific Side | 0.86 | 0.48 | 1.54 | 0.62 |
| Session | 0.99 | 0.76 | 1.3 | 0.97 |
| Age | 0.82 | 0.51 | 1.33 | 0.43 |
| Sex | 7.36 | 0.87 | 62.35 | 0.07 |

| **Table S4.** | | | | |
| --- | --- | --- | --- | --- |
| *Model 2: Seesaw reorienting predictors during the first session only.* | | | | |
| **Factor** | **Odds Ratio** | **Lower CI** | **Upper CI** | **P-value** |
| Intercept | 0.02 | 0 | 0.16 | < 0.01 *** |
| Seesaw Orientation | 7.48 | 1.98 | 28.19 | < 0.01 *** |
| Starting Position | 7.48 | 1.98 | 28.19 | < 0.01 *** |
| Reward | 0.33 | 0.04 | 2.87 | 0.32 |
